# The Dangers of Bad Plumbing

**Published:** 1881-11

**Authors:** J. C. LeHardy

**Affiliations:** Savannah, Ga.


### THE DANGERS OF BAD PLUMBING.—REPORT OF A CASE ILLUSTRATING THE DANGEROUS INFLUENCES OF SEWER GAS.

By J. C. LeHARDY, M.D., Savannah, Ga.

On August the 15th ult., Ruthy R., a very interesting little girl, four years of age, was brought to my office. Upon examining her throat I found on each tonsil a gray patch, which so closely simulated a diphtheritic membrane as to lead me to give a guarded prognosis, although there existed neither enlargement of the glands nor febrile excitement. After cauterizing the patches with stick nitrate of silver, I made inquiries in regard to the condition of the water closets and house drainage, and was told by the father, a highly-educated and intelligent lawyer, that before entering the house, some two months and a half previous, he had had them examined by one of our best plumbers, who reported that they were "all right." On examination of the patient next day the patches were found smaller and thinner, and on the third day they had entirely disappeared, and I concluded that the disease must have been caused by some gastric irritation.

The following night, however, the child was taken sick and at my visit the next morning I found her in bed, with considerable fever, temperature 102½°, pulse 120, and the glands on the left side of the neck much enlarged. On the tonsils were three patches, one on the left about the size of a three-cent piece, and two smaller ones on the right.

After cauterizing these thoroughly, and ordering three grains of bismuth sub-nitrate and two grains of precipitated sulphur, to be given every four hours (the stomach being irritable), I went with the father to make a personal inspection of the water closets and all of the water and waste pipes.

Upon removing the superstructure of the water closet up stairs, we found that the four-inch waste pipe, common to the bath tub and water closet, was ventilated by a one-inch lead pipe soldered to its upper extremity—this lead pipe having two curves, one shaped like the letter S, to enable it to pass through the wall (the closet and waste pipe being inside the building) and the other a semi-circular bend, downwards, into the gutter on the roof.

It being evident that this pipe was not sufficient to ventilate the waste pipe, and the odor of sewer gas being quite perceptible in the room, the plumber was sent for at once, and on examining the upper end of the waste pipe he found a fissure on one side of the soldering, through which gas escaped with force almost sufficient to blow out a match; there was also a fissure on the opposite side of the pipe which permitted the escape of gas into the bath room. Between the bath room and the room in which the patient and her sister, an eight year old girl, slept was a door which, although kept closed and chinked, must have undoubtedly permitted gas to pass through, for to satisfy myself on this head I called up all the children, five in number, and examined them carefully, and found that those sleeping in other rooms, which had no connection with the bath room, were free from soreness of the throat, while the other inmate of the room showed an enlargement and redness of the tonsils similar to that seen in the younger sister. No patches, however, had yet formed, probably in consequence of the greater age of the child, which enabled her to resist the disease. In a short time the plumber had exchanged the one inch pipe for one of the same diameter as the drainage pipe—four inches—and at my visit that afternoon the peculiar, pungent smell which was quite perceptible in the morning had entirely disappeared. I, however, took the precaution of having the children's room disinfected by burning sulphur in it. On the following morning, the 19th, I found the disease checked, the patches somewhat smaller and the membranes undoubtedly thinner, the glands diminishing in size, the temperature and pulse lower.

Next day—all symptoms better; one of the patches entirely gone. On the 22d the little patient was entirely free from fever, the glands very much reduced in size; the tonsils were still enlarged, and in the situation of each of the two patches was an ulcer covered with a whitish muco-purulent exudation, showing a lack of proper circulation in the parts. I touched these with blue stone (sulphate of copper) and gave the child tonic doses of quinine and tincture of the chloride of iron, and by the 30th she was entirely well. The enlargement of the tonsils of the sister also disappeared about the same time.

Comparing this case with my experience of diphtheria, and with the history of other cases in which foul gases did certainly enter the house and sleeping appartments, but where, from circumstances, it could not be prevented, I am satisfied that the happy result in this case is due entirely to the early discovery and stopping of the leak in the waste pipe and the improvement in ventilation. In the general run of cases where the source of poisoning cannot be discovered, or if known cannot be removed, the disease increases and the membranes spread, usually causing the death of the child between the fifth and the seventh days, even when treated upon the most scientific plan. The history of this case was written in order to call the attention of physicians forcibly to the importance of examining for themselves and getting their patients to examine the condition of the house drainage and ventilation, and the manner of disposing of house refuse, in the country and city, when ever called upon to see a case presenting symptoms of membranous sore throat, or of any other of the numerous class of filth diseases.

